# A scientific approach to silent consciousness

**DOI:** 10.3389/fpsyg.2013.00678

**Published:** 2013-10-10

**Authors:** Bernard J. Baars

**Affiliations:** The Neurosciences InstituteLa Jolla, CA, USA

**Keywords:** consciousness, attention, voluntary control, global workspace theory, cortex

## Introduction

All mammals show the physiological correlates of waking, dreaming and deep sleep. Many contemplative traditions propose a fourth state of consciousness, “silent consciousness,” defined as consciousness without reportable contents. For example, the *Mandukya Upanishad*, one of the root texts of Vedanta philosophy, explicitly claims a fourth state of “consciousness without content.” (Sharma, 1997) The classical summary of yogic thought, Patanjali's *Yoga Sutras*, recommends “Let there be soundless repetition of (the inner mantra) OM and meditation thereon.” (Feuerstein, 1989). The classical texts of Zen Buddhism also cite consciousness without reportable contents many times (Reps and Senzaki, 1998; Hori, 2000). About a fifth of the world population is Hindu or Buddhist.

“Silent consciousness” may sound paradoxical to Western ears, but such reports are widespread in Asian, Western, Middle Eastern, and shamanic traditions. While contemplative practices are very diverse, “inner silence” is often taken to be a goal.

One common rationale is that silent consciousness exists continuously in the background of the three standard states, and that contemplative practices aim to make that background more easily accessible to the practitioner. Thus, various practice aim to “uncover” what already exists in the mind.

The resulting access to silent consciousness is often interpreted in ontological terms as a direct knowledge of metaphysical reality. However, such claims are not considered in this article, which is focused on empirical evidence.

Benson (1984) reported such experiences in naive subjects who mentally repeated an arbitrary mantra, the word “one.” Follow-up studies show a host of non-voluntary physiological effects, including changes in stress-related gene expression, alteration in O2/CO2 exchange, and changes in energy metabolism, insulin secretion and inflammatory pathways (Dusek et al., 2008; Bhasin et al., 2013).

These findings support Benson's proposal that mantra meditation evokes a neurohormonal “relaxation response,” able to counteract the HPA stress reaction homeostatically.

The exact relationship between silent consciousness and the relaxation response is not known. An empirical probe for silent consciousness is therefore described below.

### Redundancy: fading of repeated sights, words, and actions

Contemplative practices use high numbers of repetitions: An inner syllable, a vocalized chant, a repeated breathing technique, a precise body posture, a highly practiced skill like archery, a hand gesture, a whirling dance, or a martial arts movement. Advanced practitioners spend thousands of hours in repeated actions. Nevertheless, reports of boredom are rare.

Conventional science has also studied the effects of high redundancy. Gestalt psychologists explored both the ganzfeld (a featureless visual field, like a dense, bright mist) and semantic satiation (the fading of meaning after word repetition). Both are repetition effects, and require no special apparatus. They are therefore a plausible part of ancient human practices.

The ganzfeld is defined as any visual field that lacks spatial or temporal contrast. Many neurons in the visual system are contrast-sensitive, and these cells may drop down to baseline rates of firing. Visual brightness and hue therefore tend to disappear during ganzfeld “blank-outs,” while consciousness continues (Gur, 1991).

Here are some ganzfeld examples from a Kashmiri tradition called “*112 Centering Practices*.” (Reps and Senzaki, 1998).

1. In summer when you see the entire sky endlessly clear, *enter such clarity*.
2. Simply by looking into the blue sky beyond clouds, *the serenity*.

Because an unclouded sky has no spatial or temporal contrast these could be regarded as ganzfeld conditions.

Word repetition has similar effects. Children often discover that repeating a word causes its meaning to fade. Word repetition is a near-universal practice in contemplative traditions. When subjects passively perceive repeated words, they quickly perceive word transformations (Warren, 1968). Semantic satiation (fading of meaning) tends to be noticed when subjects actively repeat words.

In vision, stabilized retinal images also fade as a function of redundancy (Rucci et al., 2007).

### Automaticity of overpracticed actions

Mantra repetition has both sensory and motor components, and highly practiced sensorimotor tasks quickly become automatic and minimally conscious. fMRI studies of automatic skills show that BOLD hot spots fade from cortex after practice (Schneider et al., 1994). Nevertheless, these tasks continue to be performed with minimal conscious involvement. Thus, the neural activities supporting the action continue after subjective fading. Since synaptic efficiency has increased with learning, networks for the skill can operate with less energy. BOLD activity therefore fades along with subjective information about the overpracticed skill.

When an automatic task is changed, cortical fMRI reappears, and task details become more consciously available. Thus, renewing a mantra after fading may reinstate conscious access for some time.

In sum, repetition may fade redundant elements from consciousness.

### Near-threshold attending

Some practices focus on barely perceivable events. Some examples from Reps and Senzaki (1998):

1. (Look at) at any point in space or on a wall—until the point *dissolves*.
2. Stop the doors of senses when feeling the creeping of an ant. *Then*.
3. Or, as breath comes in, feel the sound *hh*.
4. Intone a sound audibly, then less and less audibly as feeling deepens into this silent *harmony*.

Near-threshold attending may help subjects become more attuned to silent consciousness.

### Feelings of knowing and surprise

William James wrote the classic description of a “fringe” feeling of knowing (FOK), the tip-of–the-tongue state.

“Suppose we try to recall a forgotten name. The state of our consciousness is peculiar. … It is a gap that is intensely active. A sort of a wraith of the name is in it, beckoning us in a given direction, making us at moments tingle with the sense of our closeness, and then letting us sink back without the longed-for term. If wrong names are proposed to us, this singularly definite gap acts immediately so as to negate them. They do not fit into its mold.” (James, 1890).

Feeling of knowing are very common (Mangan, 1993). Active FOK's like the tip-of-the–tongue state evoke fMRI activity in the prefrontal cortex (Maril et al., 2001).

Baars (1988) provides an extended discussion, concluding that

1. The tip-of-the-tongue (TOT) state involves a complex representation of the expected word.
2. It occupies central limited capacity, since the state is disrupted by incompatible conscious contents.
3. Yet the TOT lacks experiential qualities like color, warmth, flavor, location, texture, intensity, etc. More broadly, feelings of knowing lack figure-ground contrast, internal structure, and clear temporal boundaries.

And yet, as James (1890) emphasized, such feelings of knowing pervade our lives.

### FOKs and surprise

FOK's may become more consciously available when they are mismatched by unexpected input. If we mentally search for the word “brontosaurus” and hear “pterodactyl” instead, we quickly recognize a mismatch, even before the correct word comes to mind. Likewise, if we see a blank space for an expected________, we can often fill in the expected content in some detail. Surprising events often seem to draw attention to our own expectations. Zen Buddhism uses surprise as a mind-changing practice.

Here is a typical report from a Zen Buddhist magazine:

“A long, thin, flat hardwood stick, the kyosaku was marched up and down the aisles …. If anyone fell asleep during zazen (as happened more than occasionally) the monitor would pounce. Whack!… whack! One good hit on each shoulder and the wary offender was awake.” (Fischer, 1998).

Sudden shocking events can induce a sense of dissociation, a drastic change in one's normal feelings of knowing. The sudden physical blow also violates one's personal space, with consequent feelings of confusion, shock, disorientation, impulsive urges to hit back, and tensing for another shock.

Zen koans also challenge expectations. A famous example is the koan “What is your original face before your mother and father were born?” Students are told to ponder such questions over and over again. Setttled answers are discouraged. Zen koans are not supposed to be resolved; the sense of paradox is itself an object of contemplation.

A somewhat different view is that long contemplation on koans is designed to interfere with the flow of ordinary conscious contents—inner speech, sensory perception, imagery and the like—so that the silent background of consciousness becomes more accessible.

For example,

A student asked Master Yun-Men (A.D. 949)

“Not even a thought has arisen (in consciousness); is there still a fault or not?”

Master replied, “*Mount Sumeru!*”

“Buddha preached for forty-nine years, but his tongue never moved,” the master Gensha said.

A monk asked Ummon, “What is the kind of talk that transcends Buddhas and Patriarchs?”

Ummon replied: “*Rice cake!*”

Notice that Zen masters do not give answers but perform interventions, constantly mismatching the student's expectations. According to Hori (2000) “*koans* are also understood as pointers to an unmediated “pure consciousness,” devoid of cognitive activity.”

## Gap matching as a probe for silent consciousness

In novices, silent moments are said to be experienced separately from the normal flow of conscious contents, like a gap in the flow of conscious sensations, images, feelings and inner speech. Silent moments might therefore be tested experimentally with a gap-detection task.

Endogenous silences may inhibit external sounds for a brief time, as is known to occur during sleep and after some hypnotic suggestions. If meditating subjects report silent gaps when there are none, they might be reporting a moment of endogenous silence. False positives in gap detection could therefore pinpoint a time window for recording neural correlates of silent consciousness (He et al., 2013). These effects could be enhanced with binaurally fused sound, commonly experienced in the center of the head.

Scalp EEG can rule out microsleeps, which have a distinctive EEG, MEG and BOLD signature (Poudel et al., 2012; Garcés Correa et al., 2013; etc.).

### Absorption and pleasure

Absorption and pleasure are often reported during silent states. Absorption is exclusive conscious engagement with one stream of thought. Silences are also described as “blissful,” adding a hedonic dimension (e.g., Reps and Senzaki, 1998). The closest analogue seems to be the notion of delight in mathematical beauty (Strogatz, 2012). These aspects deserve more study.

## A brain hypothesis

Conscious experience is believed to involve widespread cortico-thalamic oscillations in the 4–12 Hz range, modulated by higher frequency waveforms up to 200 Hz (Baars et al., 2013).

Silent consciousness may therefore correspond to increased theta-alpha power, spreading in cortex with minimal higher “content” frequencies, as has been reported during contemplative techniques.

## Summary and conclusions

Contemplative practices have been known for centuries. Western skeptics may attribute such practices to supernatural beliefs, but a better analogy may be to music training, where generations of adepts pass on a high-level skill. Music training is not arbitrary, but aims to produce precise sounds for emotional effect. Contemplative training may not be arbitrary either. They seem designed to evoke certain conscious experiences, particularly “consciousness without content.” We suggest a principled method to study the psychophysics of momentary silent consciousness.
